# Genetic and Phenotypic Variability in Chinese Patients With Branchio-Oto-Renal or Branchio-Oto Syndrome

**DOI:** 10.3389/fgene.2021.765433

**Published:** 2021-11-15

**Authors:** Haifeng Feng, Hongen Xu, Bei Chen, Shuping Sun, Rongqun Zhai, Beiping Zeng, Wenxue Tang, Wei Lu

**Affiliations:** ^1^ Department of Otorhinolaryngology-Head and Neck Surgery, The First Affiliated Hospital of Zhengzhou University, Zhengzhou, China; ^2^ Center for Applied Precision Medicine, The Second Affiliated Hospital of Zhengzhou University, Zhengzhou, China; ^3^ Precision Medicine Center, Academy of Medical Science, Zhengzhou University, Zhengzhou, China; ^4^ Henan Institute of Medical and Pharmaceutical Sciences, Zhengzhou University, Zhengzhou, China

**Keywords:** EYA1 gene, SIX1 gene, branchio-oto-renal syndrome, whole-exome sequencing, hearing rehabilitation

## Abstract

**Background:** Branchio-oto-renal syndrome (BOR) and branchio-oto syndrome (BOS) are rare autosomal dominant disorders defined by varying combinations of branchial, otic, and renal anomalies. Here, we characterized the clinical features and genetic etiology of BOR/BOS in several Chinese families and then explored the genotypes and phenotypes of BOR/BOS-related genes, as well as the outcomes of auditory rehabilitation in different modalities.

**Materials and Methods:** Probands and all affected family members underwent detailed clinical examinations. Their DNA was subjected to whole-exome sequencing to explore the underlying molecular etiology of BOR/BOS; candidate variants were validated using Sanger sequencing and interpreted in accordance with the American College of Medical Genetics guidelines. In addition, a literature review concerning *EYA1* and *SIX1* alterations was performed to explore the genotypes and phenotypes of BOR/BOS-related genes.

**Results:** Genetic testing identified the novel deletion (c.1425delC, p(Asp476Thrfs*4); NM_000,503.6), a nonsense variant (c.889C > T, p(Arg297*)), and two splicing variants in the *EYA1* gene (c.1050+1G > T and c.1140+1G > A); it also identified one novel missense variant in the *SIX1* gene (c.316G > A, p(Val106Met); NM_005,982.4). All cases exhibited a degree of phenotypic variability between or within families. Middle ear surgeries for improving bone-conduction component hearing loss had unsuccessful outcomes; cochlear implantation (CI) contributed to hearing gains.

**Conclusion:** This is the first report of BOR/BOS caused by the *SIX1* variant in China. Our findings increase the numbers of known *EYA1* and *SIX1* variants. They also emphasize the usefulness of genetic testing in the diagnosis and prevention of BOR/BOS while demonstrating that CI for auditory rehabilitation is a feasible option in some BOR/BOS patients.

## Introduction

Branchio-oto-renal syndrome-1 (BOR1; OMIM#113650), also known as Melnick–Fraser syndrome, is a rare autosomal dominant affected family members disease with an incidence of approximately one in 40,000; it affects 2% of profoundly deaf children (Melnick et al., 1975; Fraser et al., 1980). BOR exhibits a variable spectrum of clinical manifestations that are mainly characterized by the presence of branchial cleft fistulae or cysts, preauricular pits, ear malformations, and hearing loss, along with renal malformations of varying severities (Fraser et al., 1978). In some instances, patients exhibit symptoms similar to those of BOR, with the exception of renal anomalies; they are diagnosed with branchio-oto syndrome-1 (BOS1; OMIM#602588) or branchio-oto syndrome-3 (BOS3; OMIM#608389). The clinical diagnosis of BOR/BOS follows a set of criteria proposed by Chang et al. (2004). Diagnosis of BOR/BOS can be made with at least three major criteria, two major and at least two minor criteria, or one major criterion with at least one first-degree affected family member.

BOR/BOS has marked genetic heterogeneity, and the exact pathogenesis remains unknown in more than half of affected patients. Three genes are currently considered to be associated with this condition: *EYA1* (OMIM #601653), *SIX1* (OMIM #601205), and *SIX5* (OMIM #600963) (Abdelhak et al., 1997; Ruf et al., 2004; Hoskins et al., 2007). Pathogenic variants in the *EYA1* gene, the human homolog of the *Drosophila* “eyes absent” gene, were recognized as a major genetic cause of BOR/BOS; they affect 40% of BOR/BOS patients (Chang et al., 2004). The *SIX1* gene, also known as sine oculis homeobox homolog 1, encodes a transcription factor (Six1) that functions as a DNA-binding protein in combination with Eya1, leading to 3.0–4.5% of BOR/BOS cases (Ruf et al., 2004; Wang et al., 2018). The role of the *SIX5* gene, the sine oculis homeobox homolog 5, in BOR/BOS is controversial; few variants have been reported (Hoskins et al., 2007; Krug et al., 2011). The *EYA1* and *SIX1* genes are co-expressed in the developing kidney and ear, beginning during the emergence of basal plates (Xu et al., 1999). Eya1 is epistatic to Six1, and its function is dependent on interaction with Six1. Eya1 does not have direct DNA-binding ability. Instead, it functions as a transcription co-activator and interacts with Six1, thus providing a molecular mechanism for activation of specific target genes that modulate precursor cell proliferation, survival, and differentiation during multiple types of organogenesis (Li et al., 2003; Ruf et al., 2004). In the absence of this interaction, the transcriptional activation of downstream targets required for the development of the branchial, otic, and renal systems is diminished. Functional studies have confirmed that genetically defective *EYA1* and *SIX1* mice exhibit symptoms similar to BOR/BOS (Xu et al., 1999; Ando et al., 2005).

BOR/BOS is a common form of syndromic hearing loss. Hearing impairment is the most common clinical feature, present in 98.5% of affected individuals; other common features include preauricular pits or tags (83.6%), branchial fistulae or cysts (68.5%), renal anomalies (38.2%), and external auditory canal stenosis (31.5%) (Chang et al., 2004). The forms of hearing loss can be mixed (50%), conductive (30%), or sensorineural (20%), ranging in severity from mild to profound (Abdelhak et al., 1997). Most patients with BOR/BOS present with morphological abnormalities of the middle and inner ear (Chen et al., 1995; Propst et al., 2005), prompting surgeons to explore auditory rehabilitation modalities. Some studies have used middle ear exploratory tympanotomy and ossicular reconstruction to improve mixed and conductive hearing difficulties. However, the postoperative hearing gains have been unsatisfactory (Propst et al., 2005; Song et al., 2013). With the rise of auditory implant technology, cochlear implantation (CI) has provided satisfactory results in some patients with syndromic deafness (e.g., BOR/BOS) (Bajaj et al., 2012).

Here, we screened for *EYA1*, *SIX1*, and *SIX5* variants in patients with BOR or BOS and then investigated the affected individuals’ clinical manifestations and auditory rehabilitation outcomes. In addition, we analyzed the genotypes and phenotypes of BOR/BOS caused by *EYA1* variants through a literature review.

## Materials and Methods

### Patient Recruitment and Clinical Examinations

In this study, five individuals clinically diagnosed with BOR or BOS were recruited at the Affiliated Hospital of Zhengzhou University. They underwent family history inquiries and detailed physical examinations. Audiological assessment comprised pure-tone audiometry to estimate the extent of hearing loss. Objective audiometry was used for pediatric patients, including auditory brainstem response, auditory steady-state response, and distortion product otoacoustic emissions. Radiological work-up comprised temporal bone thin-section computed tomography and/or magnetic resonance imaging to analyze the middle and inner ear morphologies. Serum creatinine, urea, and renal ultrasonography analyses were performed to screen for renal abnormalities. Written informed consent was obtained from all participating individuals or their guardians prior to enrollment in the study. The project was approved by the Affiliated Hospital of Zhengzhou University (reference number: 2018008), and all procedures were performed in compliance with the Declaration of Helsinki.

### Genetic Examinations and Sanger Sequencing

Peripheral venous blood of the affected individuals was collected for genetic sequencing if available. The procedures for DNA extraction, fragmentation, library construction, targeted enrichment, and sequencing were identical to the approaches used in previous studies (Pan et al., 2020). After sequencing adaptors and inferior reads had been eliminated from raw data, clean reads were mapped to the human reference genome (version GRCh37) using Burrows-Wheeler Aligner (version 0.7.17-r1188). Duplicate reads were flagged by Sambamba (version 0.6.6) (Tarasov et al., 2015). Single-base variations and small insertions or deletions were investigated with the Genome Analysis Toolkit version four HaplotypeCaller (DePristo et al., 2011). Variant annotation, filtering, and interpretation were performed as described previously (Oza et al., 2018; Pan et al., 2020). To validate candidate variants detected by whole-exome sequencing, polymerase chain reaction amplification and Sanger sequencing were performed. Amplified polymerase chain reaction products were purified by a polymerase chain reaction purification kit (LifeSciences, Hangzhou, China) and then sequenced using the SeqStudio Genetic Analyzer (Applied Biosystems/Life Technologies, Carlsbad, CA, United States). Variant nomenclature was based on *EYA1* canonical transcript NM_000,503.6 and *SIX1* canonical transcript NM_005,982.4.

### Literature Review and Statistical Tests

Studies spanning 1975–2021 were retrieved using NCBI PubMed and the Human Gene Mutation database with “*EYA1*,” “*SIX1*,” and “branchio-oto-renal (BOR) syndrome” as the keywords. First, the *EYA1* and *SIX1* alterations were summarized. Phenotypic analysis was then performed, focusing on *EYA1* alterations that occurred in East Asian populations. When appropriate, testing for difference in proportions was carried out using either the chi-square or Fisher’s exact test. All tests were two-sided, and *p*-values lower than 0.05 were considered significant.

## Results

### Clinical Characteristics

**FIGURE 1:**
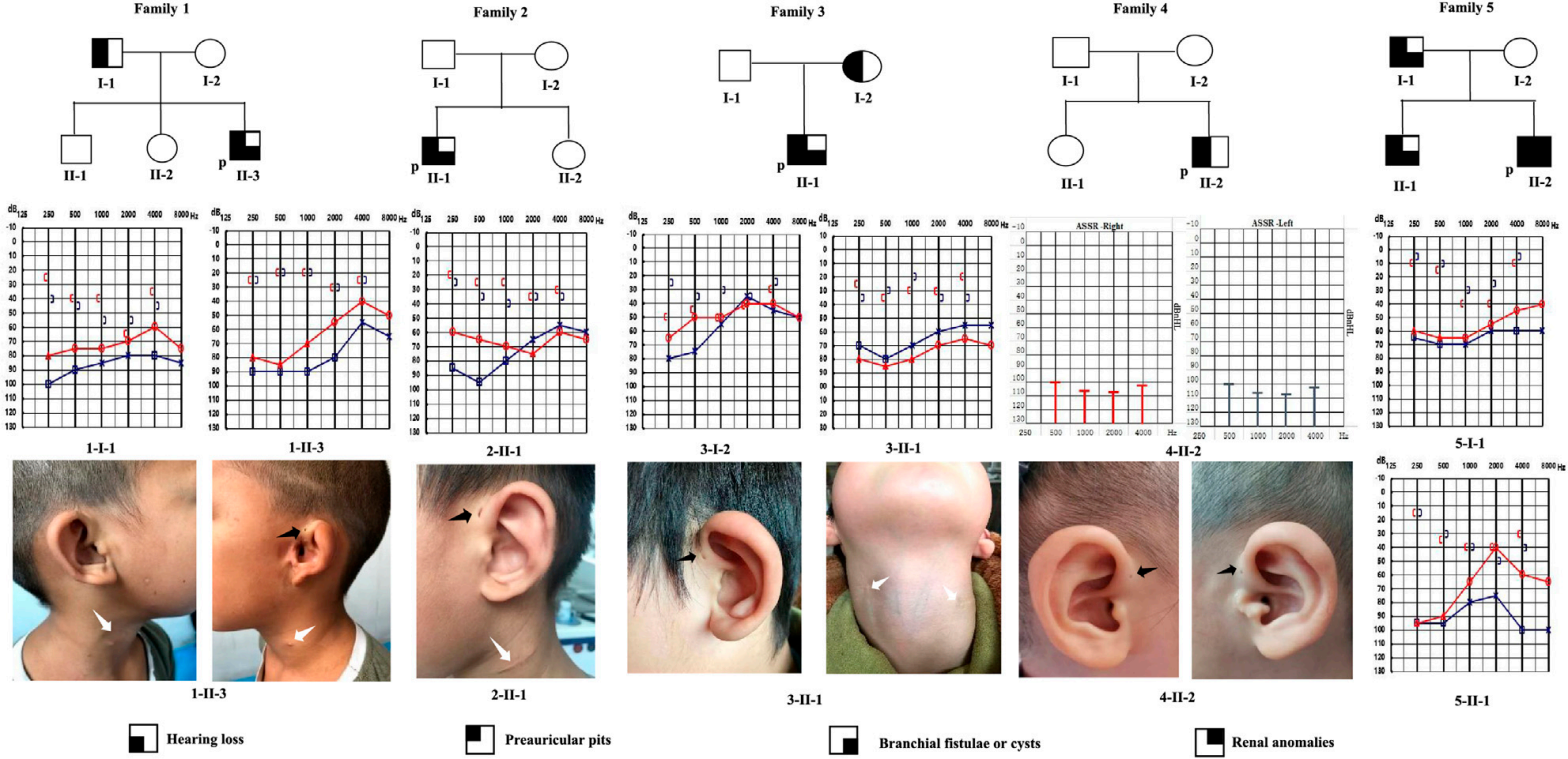
Pedigrees of five families with BOR or BOS. Audiograms (pure-tone audiometry or auditory steady-state response) and medical photographs of patients are shown. P: proband; black arrow: preauricular pits; white arrow: branchial cleft fistulae or scar.

We identified nine patients from five families with BOR or BOS, using the criteria established by Chang et al. (2004). The genealogies of the enrolled families are illustrated in Figure 1. Detailed phenotypic features are shown in Table 1. Eight patients were diagnosed with BOS; one patient was diagnosed with BOR. Of the eight patients with BOS, five showed the typical triad of BOS (preauricular pits, branchial fistulae, hearing loss); the remaining three had preauricular pits and hearing loss, without branchial fistulae or cysts. Proband 1-II-3 also had auricle deformity accompanied by preauricular tags and external auditory canal stenosis in the left ear; proband 4-II-2 had mild bilateral microtia and left preauricular tags. Renal ultrasonography showed no abnormalities in the kidneys of eight patients with respect to size, architecture, and origination; no other positive findings were noted. Proband 5-II-2, a 3-year-old, was diagnosed with BOR at 2 months old. Medical records showed that the patient had the abovementioned triad and bilateral auricle deformity. Renal ultrasonography showed left-sided renal hypoplasia and hydronephrosis, along with pyelo-ureteral separation. For radiological work-up, seven patients underwent temporal bone computed tomography. All seven patients exhibited various abnormal configurations of the middle and/or inner ear, such as deformed ossicular chain, hypoplastic cochlea, dysplastic semicircular canals, dilated internal auditory canals, or enlarged vestibular aqueduct.

**TABLE 1:** Detailed clinical features and genetic analysis of five families.

Family	Patient	B	P	D, right/left	R	CT scans	DNA	Protein	Diagnosis	ACMG evidence	ACMG classification
1	Proband	+	+	+, m/m	−	NEAC, OA, SC, CH, DIAC	EYA1:c.1050+1G > T	−	BOS	PVS1, PM2, PP3, PP4	Pathogenic
	Father	−	+	+, m/m	−	NA	EYA1:c.1050+1G > T	−	BOS
2	Proband	+	+	+, m/m	−	OA, CH, DIAC, EV, SC, EVA	EYA1:c.1140+1G > A	−	BOS	PVS1, PM2, PP3, PP4	Pathogenic
3	Proband	+	+	+, c/c	−	OC	EYA1:c.889C > T	p.(Arg297*)	BOS	PVS1, PM2, PP3, PP4	Pathogenic
	Mother	−	+	+, s/m	−	OA, CH, DIAC, EVA	EYA1:c.889C > T	p.(Arg297*)	BOS		
4	Proband	−	+	+, m/m	−	OA, SC, CH, DIAC	SIX1:c.316G > A	p.(Val106Met)	BOS	PM2, PM5, PM6, PP3, PP4	Likely pathogenic
5	Proband	+	+	+, NA	+	NA	EYA1:c.1425delC	p.(Asp476Thrfs*4)	BOR	PVS1, PM2, PP3, PP4	Pathogenic
	Brother	+	+	+, m/m	−	NEAC, OA	EYA1:c.1425delC	p.(Asp476Thrfs*4)	BOS
	Father	+	+	+, m/m	−	OA, EV, CH	NA	NA	BOS

*EYA1,* canonical transcript NM_000503.6; *SIX1,* canonical transcript NM_005,982.4; D, hearing loss; P, preauricular pits; B, branchial fistulae or cysts; R, renal anomalies; m, mixed; c, conductive; s, sensorineural; OA, ossicular anomaly; EVA, enlarged vestibular aqueduct; CH, cochlear hypoplasia; NEAC, narrowed external auditory canal; DIAC, dilated internal auditory canal; EV, enlarged vestibule; SC, abnormal semicircular canal; NA, not available.

### Bioinformatics Analysis Identifies *EYA1* and *SIX1* Variants

Probands from five families underwent whole-exome sequencing, yielding 17.69, 13.35, 12.42, 11.65, and 9.75 Gbp of raw data. The mapping rate of sequencing reads to the human reference genome was >99%. Mean sequencing depths for the targeted region were approximately 100-fold; more than 95% of the regions were covered by at least 20-fold. We investigated the coverage statistics for genes known to be associated with BOR or BOS: *EYA1*, *SIX1*, and *SIX5*. Regions with poor coverage for whole-exome sequencing (less than 20-fold) were subjected to additional polymerase chain reaction and Sanger sequencing, improving the reliability of sequencing results. After variant calling and annotation had been performed, standalone benign variants were filtered out based on the criteria specified by the Clinical Genome Resource Sequence Variant Interpretation Working Group (Ghosh et al., 2018). Subsequently, we screened in the five probands for variants in the *EYA1*, *SIX1*, and *SIX5* genes. We identified four candidate variants in the *EYA1* gene (c.1050+1G > T; c.1140+1G > A; c.889C > T, p.(Arg297*); c.1425del, p.(Asp476Thrfs*4)) and one candidate variant in the *SIX1* gene (c.316G > A, p.(Val106Met)). No *SIX5* variants were detected in any of the affected individuals.

**FIGURE 2:**
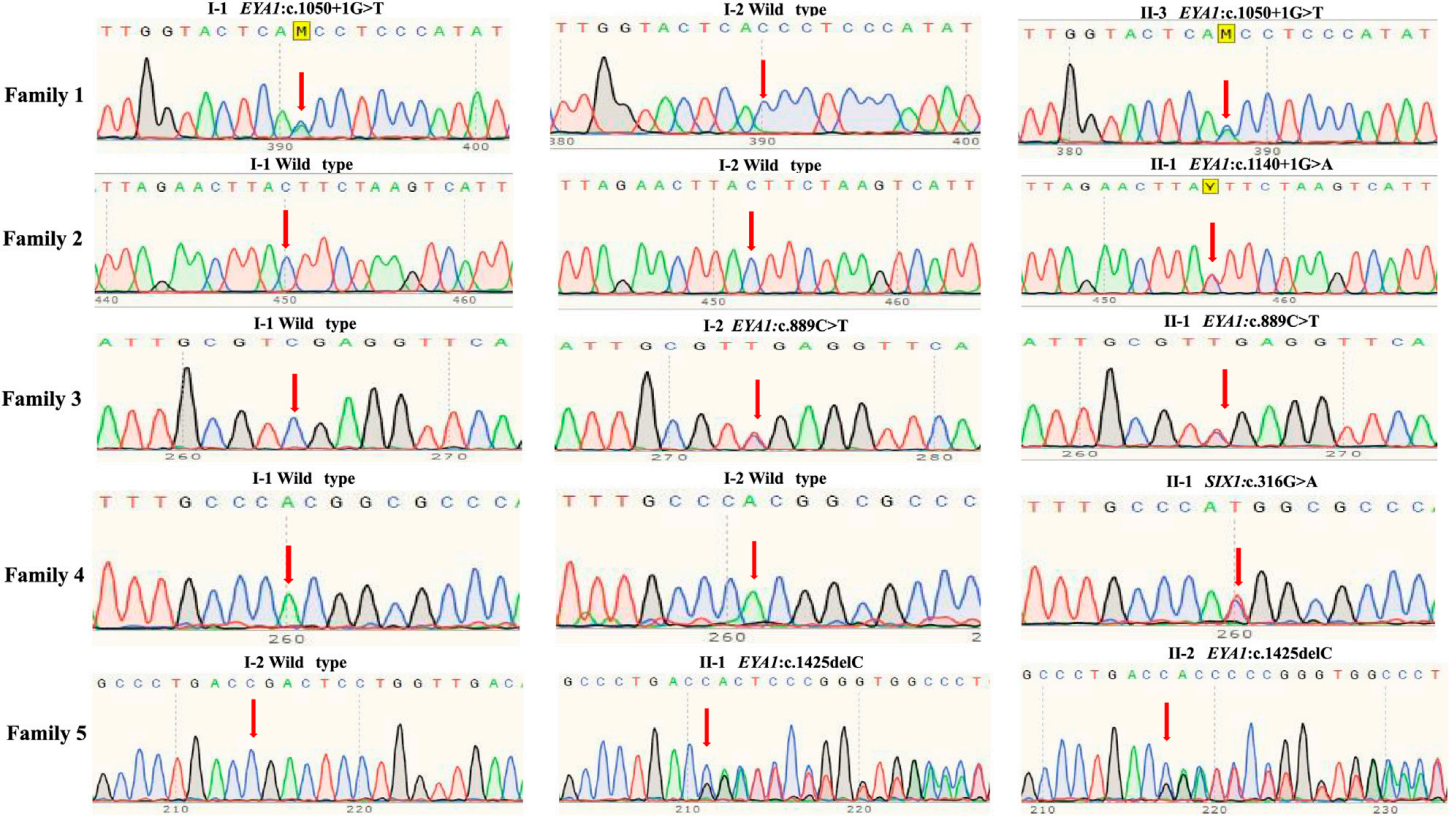
Sanger sequencing of five unrelated families with BOR or BOS. Normal family members harbored no mutations in *EYA1* or *SIX1* gene.

To confirm candidate variants and test for co-segregation, we performed Sanger sequencing (Figure 2). The *SIX1*:c.316G > A and *EYA1*:c.1140+1G > A variants in the probands were absent from both of their parents, which confirmed that these were *de novo* variants. The *EYA1*:c.1050+1G > T and *EYA1*:c.889C > T variants were confirmed to originate in the affected father and mother, respectively. We could not confirm whether the *EYA1*:c.1425del variant was from the patient’s affected father because peripheral blood was not available; however, this variant was also identified in the patient’s affected brother. The c.1425del variant of *EYA1* and c.316G > A variant of *SIX1* were not found in public databases such as gnomAD, ClinVar, and the Human Gene Mutation database, indicating that these comprised novel variants. The novel c.1425del variant in exon 15 of *EYA1* changed the arginine at codon 476 to threonine, and the C-to-T transversion at 889 in exon 10 led to the creation of a stop codon. Both were predicted to cause premature truncation of the protein. c.1050+1G > T and c.1140+1G > A variants of *EYA1* were located at canonical splice sites. The splicing effects of both variants were further predicted by SpliceAI (Jaganathan et al., 2019) and dbscSNV (Jian et al., 2014). Both variants were expected to cause aberrant splicing with donor loss scores >0.99 (SpliceAI) and ADA and RF scores >0.9 (dbscSNV). Three *EYA1* variants (c.1050+1G > T, c.1140+1G > A, c.889C > T) were previously reported in several patients with BOR or BOS from different countries (Rickard et al., 2000; Fukuda et al., 2001; Orten et al., 2008; Song et al., 2013; Unzaki et al., 2018). The c.316G > A variant of *SIX1* led to a valine-to-methionine substitution, co-segregating with the phenotype in this Chinese BOS family, and its REVEL score is 0.836. Based on the American College of Medical Genetics sequence variant interpretation guidelines, four variants (c.1050+1G > T, c.1140+1G > A, c.889C > T, and c.1425del) were classified as pathogenic; the remaining variant (c.316G > A) was classified as likely pathogenic. The American College of Medical Genetics evidence for variant interpretation is shown in Table 1.

### Auditory Rehabilitation

All affected individuals had hearing loss of varying magnitudes and presented with various forms (mixed in 15 ears, conductive in two ears, and sensorineural in one ear). In proband 1-II-3, air-bone gap persistence led to exploratory tympanotomy in the left ear before the final diagnosis. Stapedotomy and stapes prosthesis insertion were performed on the ipsilateral side because malformation, malposition, and fixation of the stapes were found. However, the air-bone gap was not narrowed. The soft-banded bone-conduction hearing aids (SOPHNON-1) trial showed over 30 dB of hearing gains. In proband 5-I-1, computed tomography revealed malformed ossicles that were surrounded by granulation tissue in the right ear. The patient was fitted with an ossicular replacement prosthesis after inflammation had subsided. Middle ear surgeries did not yield the expected results, according to self-reporting data. Proband 2-II-1 (age 7) exhibited mixed hearing loss for 3 years, which was aggravated by respiratory infections. The patient was diagnosed with otitis media with effusion; tympanostomy tube insertion and exploratory tympanotomy were performed. Ossiculoplasty was not performed because of the poor surgical outcome; the patient was then fitted for air-conduction hearing aids. Proband 4-II-2 exhibited bilateral profound deafness, which was not improved by hearing aids. Otologists made the decision to undergo CI at 1 year old in conjunction with the patient’s parents. Postoperative follow-up showed that the CI surgery was successful and had provided considerable hearing improvement (Supplementary Figure S1).

### Genotype and Phenotype Analysis

**FIGURE 3:**
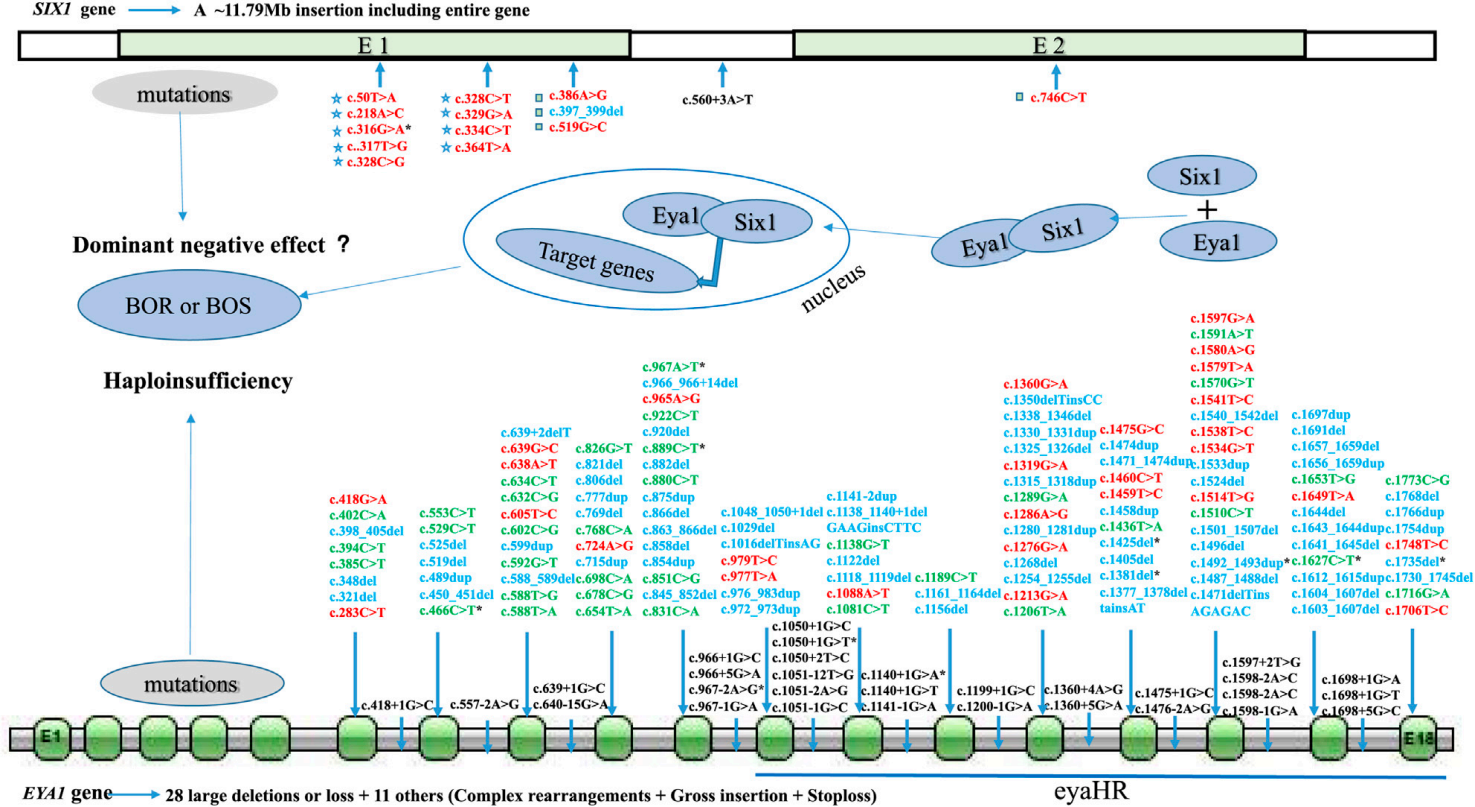
Mutational spectrum of BOR/BOS-related genes (*EYA1* and *SIX1*). **E**: exon; red: missense; green: nonsense; blue: frameshift; black: splicing site; pentagram: SIX domain of Six1 protein; square: homeodomain of Six1 protein; asterisk: variants in Chinese patients.

**FIGURE 4:**
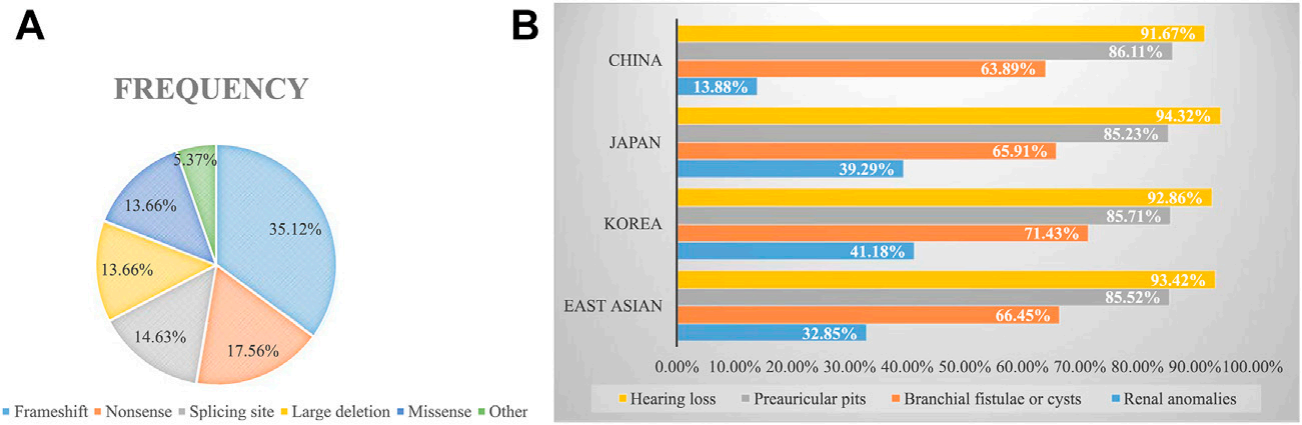
**(A)** Frequencies of variant types in the *EYA1* gene. **(B)** Estimated prevalence of clinical phenotypes among BOR/BOS patients with *EYA1* variant.

To explore the mutational spectrum of BOR/BOS-related genes, we summarized the reported variants of the *EYA1* and *SIX1* genes shown in Figure 3. Variants in the *EYA1* gene were found in various forms; frameshift was the most common type, followed by nonsense, splicing, large deletion, and missense (Figure 4A). In the *SIX1* gene, the most commonly reported variant was missense (12/15) (Figure 3). The results exhibited genetic variability. Subsequently, we analyzed the phenotypic characteristics of BOR/BOS patients with the *EYA1* variant in East Asian populations (Table 2 and Figure 4B). The results indicated that hearing loss is the most common symptom, with an estimated prevalence of 93.42%, followed by preauricular pits (85.52%), branchial fistulae or cysts (66.45%), and renal anomalies (32.85%). A small number of cases have been reported in China (Table 2); the morbidities were hearing loss (91.67%), preauricular pits (86.11%), branchial fistulae or cysts (63.89%), and renal anomalies (13.88%) (Figure 4B). The incidence of each phenotype in Chinese patients was further compared with those in Japanese and Korean patients. These proportions in hearing loss, preauricular pits, and branchial fistulae were not significantly different from that observed in patients from Japan and Korea (*p* = 0.904, *p* = 1.000, *p* = 0.816). The proportion of renal anomalies was statistically different (*p* = 0.019), and cases with renal phenotypes in China seem to be scarce, compared with Japan (*p* = 0.009) and Korea (*p* = 0.038), indicating that patients may present mainly with BOS in China.

**TABLE 2:** Genotypes and phenotypes of patients with BOR/BOS caused by *EYA1* variants in East Asian populations.

Country	DNA change	Location	Protein change*	Phenotype (B, D, P, R)	Family history	References
China	c.466C > T	Exon 7	p.(Gln156*)	B (2/3), D (2/3), P (3/3)	F	Wang et al. (2012)
—	c.889C > T	Exon 10	p.(Arg297*)	B (1/2), D (2/2), P (1/2)	F	This study
—	c.967–2A > G	Intron 10	Splicing site	B (3/3), D (3/3), P (3/3)	F	Chen et al. (2019)
—	c.967A > T	Exon 11	p.(Arg323*)	B (3/6), D (6/6), P (5/6), R (1/6)	F	Wang et al. (2018)
—	c.1050+1G > T	Intron 11	Splicing site	B (1/2), D (2/2), P (2/2)	F	This study
—	c.1140+1G > A	Intron 12	Splicing site	B, D, P	S	This study
—	c.1381del	Exon 15	p.(Arg461Glyfs*7)	B, P, R	S	Li et al. (2018)
—	c.1425del	Exon 15	p.(Asp476Thrfs*4)	B (2/3), D (3/3), P (3/3), R (1/3)	F	This study
—	c.1492_1493dup	Exon 16	p.(Ile499Phefs*33)	D (4/4), P (4/4)	F	Chen et al. (2019)
—	c.1627C>T	Exon 17	p.(Gln543*)	B (4/4), D (4/4), P (4/4)	F	Han et al. (2021)
—	c.1735del	Exon 18	p.(Asp579fs*60)	B (1/3), D (2/3), P (2/3)	F	Wang et al. (2012)
—	Entire deletion	Exon 1–18	Loss protein	B (3/3), D (3/3), P (1/3), R (1/3)	F	Men et al. (2020)
—	Entire deletion	Exon 1–18	Loss protein	B, D, P, R	S	Chen et al. (2014)
Japan	c.418+1G > C	Intron 6	Splicing site	B, D,R	S	Unzaki et al. (2018)
—	c.588T > A	Exon 8	p.(Tyr196*)	D, P, R	S	Okada et al. (2006)
—	c.588T > G	Exon 8	p.(Tyr196*)	D (2/2), P (2/2)	F	Ideura et al. (2019)
—	c.632C > G	Exon 8	p.(Ser211*)	B (2/4), D (3/4), P (4/4), R (1/4)	F	Uno et al. (2004)
—	c.634C > T	Exon 8	p.(Gln212*)	B (2/2), D (2/2), P (2/2), R (1/2)	F	Unzaki et al. (2018)
—	c.678C > G	Exon 9	p.(Tyr226*)	B (2/3), D (3/3), P (3/3), R (1/3)	F	Usami et al. (1999)
—	c.698C > A	Exon 9	p.(Ser233*)	B, D, P	S	Unzaki et al. (2018)
—	c.724A > G	Exon 9	p.(Ser242Gly)	D (2/2), P (1/2)	F	Yashima et al. (2003)
—	c.880C > T	Exon 10	p.(Arg294*)	B, D, P, R	S	Unzaki et al. (2018)
—	c.889C > T	Exon 10	p.(Arg297*)	B (11/11), D (11/11), P (11/11)	F	Fukuda et al. (2001)
—	c.922C > T	Exon 10	p.(Arg308*)	B, D, R	S	Unzaki et al. (2018)
—	c.1050+2T > C	Intron 11	Splicing site	D, P, R	S	Unzaki et al. (2018)
—	c.1051–2A > G	Intron 11	Splicing site	B (2/3), D (3/3), P (3/3), R (1/3)	F	Okada et al. (2006)
—	c.1051G > T	Exon 12	p.(Asp351Tyr)	B (1/3), D (3/3), P (3/3), R (1/3)	F	Okada et al. (2006)
—	c.1122delA	Exon 12	p.(Leu374Phefs*6)	B (2/2), D (2/2), P (2/2), R (1/2)	F	Unzaki et al. (2018)
—	c.1140+1G > A	Intron 12	Splicing site	B (3/3), D (3/3), P (3/3), R (2/3)	F	Unzaki et al. (2018)
—	c.1161_1164del	Exon 13	p.(Ile387Metfs*12)	B (1/2), D (2/2), P (1/2), R (1/2)	F	Unzaki et al. (2018)
—	c.1206T > A	Exon 13	p.(Tyr402*)	D, P, R	S	Okada et al. (2006)
—	c.1254_1255del	Exon 14	p.(Cys419Phefs*32)	B, D, P, R (NA)	S	Ideura et al. (2019)
—	c.1276G > A	Exon 14	p.(Gly426Ser)	B, D, R	S	Namba et al. (2001)
—	c.1286A > G	Exon 14	p.(Asp429Gly)	B, D, P	S	Namba et al. (2001)
—	c.1289G > A	Exon 14	p.(Trp430*)	B (2/2), D (2/2), P (2/2), R (2/2)	F	Unzaki et al. (2018)
—	c.1319G > A	Exon 14	p.(Arg440Gln)	B (5/8), D (7/8), P (6/8), R (4/8)	F/S	Unzaki et al. (2018)
—	c.1501_1507del	Exon 16	p.(Thr501Leufs*15)	B (3/4), D (4/4), P (2/4)	F	Shimasaki et al. (2004)
—	c.1598-1G > A	Intron 16	Splicing site	B, D, P, R	S	Okada et al. (2006)
—	c.1643_1644dup	Exon 17	p.(Val549Lysfs*7)	B, D, P, R	S	Unzaki et al. (2018)
—	c.1730_1745del	Exon 18	p.(His577Profs*57)	D (3/3), P (3/3), R (2/3)	F	Unzaki et al. (2018)
—	c.1766dup	Exon 18	p.(Glu590Glyfs*42)	B (5/5), D (4/5), P (5/5), R (1/5)	F	Matsunaga et al. (2007)
—	Partial deletion	Exon 4–7	Truncated protein	D, P, R	S	Morisada et al. (2010)
—	Partial deletion	Exon 10–18	Truncated protein	B (2/2), D (2/2), P (2/2)	F	Unzaki et al. (2018)
—	Partial deletion	Exon 2–3	Truncated protein	D (3/3), P (1/3), R (1/3)	F	Unzaki et al. (2018)
—	Partial deletion	Exon 2–12	Truncated protein	B, D, P	S	Unzaki et al. (2018)
—	Partial deletion	Exon 12	Truncated protein	D, P, R	S	Unzaki et al. (2018)
—	Partial deletion	Exon 17	Truncated protein	B (4/4), D (3/4), P (4/4), R (2/4)	F	Unzaki et al. (2018)
—	Partial deletion	Exon 17	Truncated protein	D (2/2), R (1/2)	F	Unzaki et al. (2018)
—	Entire deletion	Exon 1–18	Loss protein	B (1/3), D (3/3), P (3/3), R (NA)	F	Ideura et al. (2019)
Korea	c.321del	Exon 6	p.(Ala107fs*133)	B (2/2), D (2/2), P (2/2), R (1/2)	F	Lee et al. (2009)
—	c.418G > A	Exon 6	p.(Gly140Ser)	B, D, P	S	Kim et al. (2014)
—	c.529C > T	Exon 7	p.(Gln177*)	B (10/10), D (9/10), P (10/10), R (4/5)	F	Lee et al. (2007)
—	c.699+5G > A	Intron 10	Splicing site	D, R (NA)	S	Song et al. (2013)
—	c.965A > G	Exon 10	p.(Glu322Gly)	D (4/4), P (4/4), R (NA)	F	Song et al. (2013)
—	c.967–2A > G	Intron 10	Splicing site	B (3/4), D (3/4), P (4/4)	F	Kwon et al. (2009)
—	c.1140+1G > A	Intron 12	Splicing site	D, P, R (NA)	S	Song et al. (2013)
—	c.1474dup	Exon 15	p.(Arg492Profs*40)	B (3/3), D (3/3), R (2/3)	F	Kim et al. (2005)
—	c.1598–2A > C	Intron 16	Splicing site	B, D, P	S	Song et al. (2013)
—	Entire deletion	Exon 1–18	Loss protein	D, P	S	Song et al. (2013)

*EYA1,* canonical transcript NM_000,503.6; *SIX1,* canonical transcript NM_005,982.4; D, hearing loss; P, preauricular pits; B, branchial fistulae or cysts; R, renal anomalies; NA, not available or not done; F, family history; S, scatter.

## Discussion

This study described nine affected individuals in five Han Chinese families with BOR or BOS. Of the nine patients, all had hearing loss (9/9) and preauricular pits (9/9), five had branchial fistulae or cysts (5/9), and only one had renal abnormalities (1/9), indicating that BOS is the major form. Genetic analysis confirmed five causative heterozygous variants in these unrelated families: c.1425delC (frameshift), c.889C > T (nonsense), c.1050+1G > T (splicing), and c.1140+1G > A (splicing) of *EYA1*, as well as c.316 > A (missense) of *SIX1*. To our knowledge, *EYA1*:c.1425delC and *SIX1*:c.316 > A have not been previously reported; c.316G > A is also the first reported *SIX1* variant in China. Our findings expand the mutational spectrum of BOR/BOS-related genes and demonstrate genetic and phenotypic variability in Chinese BOR/BOS patients.

*EYA1* and *SIX1* are key genes for mammalian organogenesis; mutations in these genes result in multiorgan malformation that can affect the branchial, ear, and renal systems. In 1997, Abdelhak and others reported novel variants in the *EYA1* gene in several families demonstrating typical manifestations of BOR/BOS (Abdelhak et al., 1997). Since then, more than 200 variants have been identified (Figure 3). The *EYA1* gene, located at 8q13.3, is a member of the *EYA* (*EYA1–4*) family; it contains 18 exons encoding a dual-function transcription factor. Similar to other EYA family members, Eya1 possesses a divergent N-terminal transactivation domain and a C-terminal EYA dephosphorylation region (eyaHR) encoded by 11–18 exons (Abdelhak et al., 1997; Li et al., 2003). Known variants in *EYA*1 mainly involve exons 6–18, especially exons 11–18, at least for now (Figure 3). These variants mainly cause premature truncation or aberrant splicing of the protein, resulting in the loss of eyaHR function. Screened *EYA1* variants in this study were predicted to interfere with this critical function. The protein Six1 encoded by the *SIX1* gene, a co-factor of Eya1, contains two evolutionarily conserved domains: a SIX domain that interacts with its co-factors and a homeodomain with DNA-binding ability (Ruf et al., 2004). Of the defects in the *SIX1* gene, Six1 functional alterations led to the failed formation of the Six1–Eya1 complex and the DNA–Six1–Eya1 complex (Ruf et al., 2004). Thus far, 14 *SIX1* variants are reportedly associated with BOR/BOS, eight of which are in the SIX domain (Figure 3). We also identified a novel missense variant (c.316G > A) in the *SIX1* gene; we speculate that this variant interferes with Six1 binding to Eya1, which has been previously reported for variants in the SIX domain (Ruf et al., 2004; Kochhar et al., 2008). Loss of function of either gene in a mouse model led to hearing loss and dysmorphic or missing kidneys, along with developmental abnormalities in other organs (Xu et al., 1999; Li et al., 2003). Our patients harboring *EYA1* or *SIX1* alterations also had hearing loss, ear deformities, or kidney problems.

BOR/BOS patients demonstrate intrafamilial and interfamilial phenotypic variability in the clinical setting, suggesting a lack of genotype–phenotype correlation. In the present study, proband 5-II-2 harboring c.1425delC exhibited all major symptoms of BOR, while patients 5-II-1 and 5-I-1 only presented with the triad of BOS, demonstrating coexistence of BOR and BOS in this family. The presence of interfamilial phenotypic variability has also been confirmed. A patient from China with the same c.1140+1G > A variant demonstrated no renal involvement; patients from Korea reportedly lack branchial fistulae; and Japanese patients have four main symptoms of BOR (Table 2). Notably, most patients carrying the same or different variants at the same position, such as c.1050+1G > T (Orten et al., 2008), c.1050+1G > C (Henriksen et al., 2004), or c.1050+2T > C (Unzaki et al., 2018), showed renal anomalies; such anomalies were not observed in our patients with the c.1050+1G > T variant. This variability is also present among patients with *SIX1* variants. Patients with c.328C > T variant exhibited distinct phenotypes in several unrelated families (Kochhar et al., 2008). In addition, c.316G > A and c.317T > G led to the substitution of amino acids at the same position to methionine and glycine, respectively (Kochhar et al., 2008). Two individuals exhibited similar symptoms of BOS. Specifically, the same or a similar variant can cause variable clinical phenotypes in BOR/BOS, while different variants can cause similar clinical phenotypes.

The mechanism by which mutations produce phenotypic variability is undefined in BOR/BOS. Known *EYA1* variants mainly comprise loss-of-function mutations; such variants typically imply haploinsufficiency through reduced gene dosage and expression (Figure 3) (Zhang et al., 2004). These results suggest that the inconsistency of phenotypes is partially influenced by the dosage effect. The Eya1 protein activates target genes controlling the development of the branchial arch, ear, or kidney only when a specific threshold is exceeded (Wang et al., 2018). Otherwise, environmental factors and genetic modifiers might also modify the phenotypes of BOR/BOS. Notably, we found that reported *SIX1* variants mainly comprise missense (12/15). In the future, further exploration of the role of the dominant-negative effect (Shah et al., 2020) is also warranted.

BOS appears to be the main manifestation in China. In 1995, a study indicated that the estimated prevalence of renal abnormality was 67% in 21 patients with BOR in the United States; renal agenesis occurred most often, followed by hypoplasia, renal dysplasia, ureteral-pelvic junction obstruction, calyceal cyst/diverticulum, caliectasis, and hydronephrosis (Chen et al., 1995). In 2004, Chang et al. reported the renal abnormality frequency of 38% based on analyses of 40 families, but no detailed renal phenotypes were described (Chang et al., 2004). In France, Kurg et al. also identified 53% of the prevalence of renal anomalies in BOR patients harboring *EYA1* variants; there is a wide range of abnormalities, namely, renal hypoplasia, multicystic kidney dysplasia, agenesis, abnormal pyelo-ureteral junction, and kidney malrotation, to name the most common (Krug et al., 2011). In our study, only one patient presented with left-sided renal hypoplasia, hydronephrosis, and pyelo-ureteral separation (1/9). We then calculated the frequency of renal malformations in East Asian populations through a literature review (Figure 4B). The frequencies were 14% in China, 39% in Japan, and 41% in Korea. Comparatively, the morbidity of renal anomalies appears to be lower and statistically different, suggesting that patients may primarily present with BOS in China. The elucidation of population differences in renal anomalies requires further exploration.

The clinical management of BOR/BOS requires individual management and multidisciplinary collaboration because of the multi-systemic symptoms. Deafness, the most prevalent manifestation of BOR/BOS, requires close attention and early treatment. Patients with BOR/BOS exhibit multiple structural malformations of the middle ear, especially ossicular malformations. Surgeons perform ossicular reconstruction to repair mixed or conductive hearing loss (Cremers et al., 1981), but the results are not always satisfactory. Our therapeutic findings for probands 1-II-3 and 5-I-1 were consistent with previous results. Thus, we presume that middle ear surgery is not optimal for patients with BOR/BOS. Hearing aids are beneficial to most patients, but this treatment should be individually tailored. Proband 1-II-3 received soft-banded bone-conduction hearing aids, while proband 2-II-1 wore air-conduction hearing aids after fitting. Their hearing abilities were substantially improved, which facilitated the fulfillment of learning and communication needs. Miyagawa et al. performed bone-anchored hearing aid implantation on a patient with BOS, which led to an improved hearing threshold (Miyagawa et al., 2015). Therefore, bone-conduction hearing device implantation may be considered when cranial bone thickness is at least 4 mm. CI may be a good option when hearing aids do not substantially improve hearing. Kameswaran et al. performed CI on a BOR patient with multiple inner ear malformations (Kameswaran et al., 2007); Bajaj et al. analyzed the surgical effect of CI in syndromic children, including children with BOR/BOS (Bajaj et al., 2012); their results indicated that CI was feasible in patients with BOR/BOS. In the present study, proband 5-II-3 underwent CI in the right ear after a comprehensive evaluation of the preoperative findings. CI can be successful through careful intraoperative operation, and the postoperative hearing ability is satisfactory with rehabilitation.

In conclusion, the symptoms of BOR or BOS in this study were attributed to *EYA1* or *SIX1* alterations. BOR/BOS exhibited some genetic and phenotypic variability. The outcomes of auditory rehabilitation reiterated that middle ear surgeries are generally unsatisfactory in patients with BOR/BOS; CI may be a feasible option when patients cannot benefit from hearing aids. Genetic testing contributes to the diagnosis and future genetic consultation; it has vital roles in therapy and intervention.

## Data Availability

All data supporting the findings of this study are available on request from the corresponding author. The pathogenic variants have been submitted to ClinVar (https://www.ncbi.nlm.nih.gov/clinvar/) with the accession number (VCV001202644, VCV001202645, VCV000854287, VCV000429912, VCV001202646).

## Supplementary Material

Supplementary Data Sheet S1
**(A)** The mapping after CI surgery (Map). **(B)** Neural response telemetry (NRT). **(C)** Behavioral audiometry.

## References

[B1] Abdelhak S. Kalatzis V. Heilig R. Compain S. Samson D. Vincent C. (1997). A Human Homologue of the Drosophila Eyes Absent Gene Underlies Branchio-Oto-Renal (BOR) Syndrome and Identifies a Novel Gene Family. Nat. Genet. 15 (2), 157–164. 10.1038/ng0297-157 9020840

[B2] Ando Z.-I. Sato S. Ikeda K. Kawakami K. (2005). Slc12a2 Is a Direct Target of Two Closely Related Homeobox Proteins, Six1 and Six4. Febs j 272 (12), 3026–3041. 10.1111/j.1742-4658.2005.04716.x 15955062

[B3] Bajaj Y. Gibbins N. Fawkes K. Hartley B. Jephson C. Jonas N. (2012). Surgical Aspects of Cochlear Implantation in Syndromic Children. Cochlear Implants Int. 13 (3), 163–167. 10.1179/1754762811y.0000000020 22334127

[B4] Chang E. H. Menezes M. Meyer N. C. Cucci R. A. Vervoort V. S. Schwartz C. E. (2004). Branchio-Oto-Renal Syndrome: The Mutation Spectrum inEYA1and its Phenotypic Consequences. Hum. Mutat. 23 (6), 582–589. 10.1002/humu.20048 15146463

[B5] Chen A. Francis M. Ni L. Cremers C. W. R. J. Kimberling W. J. Sato Y. (1995). Phenotypic Manifestations of Branchiootorenal Syndrome. Am. J. Med. Genet. 58 (4), 365–370. 10.1002/ajmg.1320580413 8533848

[B6] Chen P. Liu H. Lin Y. Xu J. Zhu W. Wu H. (2019). EYA1 Mutations Leads to Branchio-Oto Syndrome in Two Chinese Han Deaf Families. Int. J. Pediatr. Otorhinolaryngol. 123, 141–145. 10.1016/j.ijporl.2019.05.006 31102969

[B7] Chen X. Wang J. Mitchell E. Guo J. Wang L. Zhang Y. (2014). Recurrent 8q13.2-13.3 Microdeletions Associated with Branchio-Oto-Renal Syndrome Are Mediated by Human Endogenous Retroviral (HERV) Sequence Blocks. BMC Med. Genet. 15, 90. 10.1186/s12881-014-0090-9 25135225 PMC4152767

[B8] Cremers C. W. R. J. Thijssen H. O. M. Fischer A. J. E. M. Marres E. H. M. A. (1981). Otological Aspects of the Earpit-Deafness Syndrome. ORL J. Otorhinolaryngol. Relat. Spec. 43 (4), 223–239. 10.1159/000275541 6973119

[B9] DePristo M. A. Banks E. Poplin R. Garimella K. V. Maguire J. R. Hartl C. (2011). A Framework for Variation Discovery and Genotyping Using Next-Generation DNA Sequencing Data. Nat. Genet. 43 (5), 491–498. 10.1038/ng.806 21478889 PMC3083463

[B10] Fraser F. C. Ling D. Clogg D. Nogrady B. Gorlin R. J. (1978). Genetic Aspects of the BOR Syndrome-Branchial Fistulas, Ear Pits, Hearing Loss, and Renal Anomalies. Am. J. Med. Genet. 2 (3), 241–252. 10.1002/ajmg.1320020305 263442

[B11] Fraser F. C. Sproule J. R. Halal F. Optiz J. M. (1980). Frequency of the Branchio-Oto-Renal (BOR) Syndrome in Children with Profound Hearing Loss. Am. J. Med. Genet. 7 (3), 341–349. 10.1002/ajmg.1320070316 7468659

[B12] Fukuda S. Kuroda T. Chida E. Shimizu R. Usami S.-I. Koda E. (2001). A Family Affected by Branchio-Oto Syndrome with EYA1 Mutations. Auris Nasus Larynx 28 (Suppl. l), S7–S11. 10.1016/s0385-8146(01)00082-7 11683347

[B13] Ghosh R. Harrison S. M. Rehm H. L. Plon S. E. Biesecker L. G. (2018). Updated Recommendation for the Benign Stand‐Alone ACMG/AMP Criterion. Hum. Mutat. 39 (11), 1525–1530. 10.1002/humu.23642 30311383 PMC6188666

[B14] Han R. Xia Y. Liu Z. Wu S. Ye E. Duan L. (2021). A Mutation of EYA1 Gene in a Chinese Han Family with Branchio-Oto Syndrome. Medicine (Baltimore) 100 (25), e24691. 10.1097/md.0000000000024691 34160378 PMC8238333

[B15] Henriksen A. M. Tümer Z. Tommerup N. Tranebjærg L. Larsen L. A. (2004). Identification of a NovelEYA1Splice-Site Mutation in a Danish Branchio-Oto-Renal Syndrome Family. Genet. Test. 8 (4), 404–406. 10.1089/gte.2004.8.404 15684871

[B16] Hoskins B. E. Cramer C. H. Silvius D. Zou D. Raymond R. M. Orten D. J. (2007). Transcription Factor SIX5 Is Mutated in Patients with Branchio-Oto-Renal Syndrome. Am. J. Hum. Genet. 80 (4), 800–804. 10.1086/513322 17357085 PMC1852719

[B17] Ideura M. Nishio S.-y. Moteki H. Takumi Y. Miyagawa M. Sato T. (2019). Comprehensive Analysis of Syndromic Hearing Loss Patients in Japan. Sci. Rep. 9 (1), 11976. 10.1038/s41598-019-47141-4 31427586 PMC6700179

[B18] Jaganathan K. Kyriazopoulou Panagiotopoulou S. McRae J. F. Darbandi S. F. Knowles D. Li Y. I. (2019). Predicting Splicing from Primary Sequence with Deep Learning. Cell 176 (3), 535–548. e524. 10.1016/j.cell.2018.12.015 30661751

[B19] Jian X. Boerwinkle E. Liu X. (2014). In Silico Prediction of Splice-Altering Single Nucleotide Variants in the Human Genome. Nucleic Acids Res. 42 (22), 13534–13544. 10.1093/nar/gku1206 25416802 PMC4267638

[B20] Kameswaran M. Kumar R. S. A. Murali S. Raghunandhan S. Karthikeyan K. (2007). Cochlear Implantation in Branchio-Oto-Renal Syndrome - A Surgical Challenge. Indian J. Otolaryngol. Head Neck S 59 (3), 280–283. 10.1007/s12070-007-0081-7 PMC3452117 23120453

[B21] Kim H. R. Song M. H. Kim M.-A. Kim Y.-R. Lee K.-Y. Sonn J. K. (2014). Identification of a Novel Nonsynonymous Mutation of EYA1 Disrupting Splice Site in a Korean Patient with BOR Syndrome. Mol. Biol. Rep. 41 (7), 4321–4327. 10.1007/s11033-014-3303-6 24590738

[B22] Kim S. H. Shin J.-H. Yeo C.-K. Chang S. H. Park S.-Y. Cho E. H. (2005). Identification of a Novel Mutation in the EYA1 Gene in a Korean Family with Branchio-Oto-Renal (BOR) Syndrome. Int. J. Pediatr. Otorhinolaryngol. 69 (8), 1123–1128. 10.1016/j.ijporl.2005.03.003 16005355

[B23] Kochhar A. Orten D. J. Sorensen J. L. Fischer S. M. Cremers C. W. R. J. Kimberling W. J. (2008). SIX1mutation Screening in 247 Branchio-Oto-Renal Syndrome Families: A Recurrent Missense Mutation Associated with BOR. Hum. Mutat. 29 (4), 565. 10.1002/humu.20714 18330911

[B24] Krug P. Morinière V. Marlin S. Koubi V. Gabriel H. D. Colin E. (2011). Mutation Screening of the EYA1, SIX1, and SIX5 Genes in a Large Cohort of Patients Harboring Branchio-Oto-Renal Syndrome Calls into Question the Pathogenic Role of SIX5 Mutations. Hum. Mutat. 32 (2), 183–190. 10.1002/humu.21402 21280147

[B25] Kwon M.-J. Boo S. H. Kwon M.-J. Boo S. H. Kim H.-J. Cho Y.-S. (2009). A Novel Splice Site Mutation in theEYA1gene in a Korean Family with Branchio-Oto (BO) Syndrome. Acta Oto-Laryngologica 129 (6), 688–693. 10.1080/00016480802342432 18763178

[B26] Lee J. D. Kim S. C. Koh Y. W. Lee H. J. Choi S. Y. Kim U. K. (2009). A Novel Frameshift Mutation in the EYA1 Gene in a Korean Family with Branchio-Oto-Renal Syndrome. Ann. Clin. Lab. Sci. 39 (3), 303–306. 19667416

[B27] Lee K. Y. Kim S. Kim U. K. Ki C.-S. Lee S. H. (2007). Novel EYA1 Mutation in a Korean Branchio-Oto-Renal Syndrome Family. Int. J. Pediatr. Otorhinolaryngol. 71 (1), 169–174. 10.1016/j.ijporl.2006.08.023 17049623

[B28] Li G. Shen Q. Sun L. Liu H. An Y. Xu H. (2018). A De Novo and Novel Mutation in the EYA1 Gene in a Chinese Child with Branchio-Oto-Renal Syndrome. Intractable Rare Dis. Res. 7 (1), 42–45. 10.5582/irdr.2017.01075 29552445 PMC5849624

[B29] Li X. Ohgi K. A. Zhang J. Krones A. Bush K. T. Glass C. K. (2003). Eya Protein Phosphatase Activity Regulates Six1-Dach-Eya Transcriptional Effects in Mammalian Organogenesis. Nature 426 (6964), 247–254. 10.1038/nature02083 14628042

[B30] Matsunaga T. Okada M. Usami S.-I. Okuyama T. (2007). Phenotypic Consequences in a Japanese Family Having Branchio-Oto-Renal Syndrome with a Novel Frameshift Mutation in the geneEYA1. Acta Oto-Laryngologica 127 (1), 98–104. 10.1080/00016480500527185 17364338

[B31] Melnick M. Bixler D. Silk K. Yune H. Nance W. E. (1975). Autosomal Dominant Branchiootorenal Dysplasia. Birth Defects Orig. Artic Ser. 11 (5), 121–128. 1218203

[B32] Men M. Li W. Chen H. Wu J. Feng Y. Guo H. (2020). Identification of a Novel CNV at 8q13 in a Family with Branchio‐Oto‐Renal Syndrome and Epilepsy. The Laryngoscope 130 (2), 526–532. 10.1002/lary.27941 30908667

[B33] Miyagawa M. Nishio S.-y. Hattori M. Takumi Y. Usami S.-i. (2015). Germinal Mosaicism in a Family with BO Syndrome. Ann. Otol. Rhinol. Laryngol. 124 (Suppl. 1), 118s–122s. 10.1177/0003489415575062 25780253

[B34] Morisada N. Rendtorff N. D. Nozu K. Morishita T. Miyakawa T. Matsumoto T. (2010). Branchio-Oto-Renal Syndrome Caused by Partial EYA1 Deletion Due to LINE-1 Insertion. Pediatr. Nephrol. 25 (7), 1343–1348. 10.1007/s00467-010-1445-x 20130917

[B35] Namba A. Abe S. Shinkawa H. Kimberling W. J. Usami S. (2001). Genetic Features of Hearing Loss Associated with Ear Anomalies: PDS and EYA1 Mutation Analysis. J. Hum. Genet. 46 (9), 518–521. 10.1007/s100380170033 11558900

[B36] Okada M. Fujimaru R. Morimoto N. Satomura K. Kaku Y. Tsuzuki K. (2006). EYA1 and SIX1 Gene Mutations in Japanese Patients with Branchio-Oto-Renal (BOR) Syndrome and Related Conditions. Pediatr. Nephrol. 21 (4), 475–481. 10.1007/s00467-006-0041-6 16491411

[B37] Orten D. J. Fischer S. M. Sorensen J. L. Radhakrishna U. Cremers C. W. R. J. Marres H. A. M. (2008). Branchio-oto-renal Syndrome (BOR): Novel Mutations in theEYA1gene, and a Review of the Mutational Genetics of BOR. Hum. Mutat. 29 (4), 537–544. 10.1002/humu.20691 18220287

[B38] Oza A. M. DiStefano M. T. Hemphill S. E. Cushman B. J. Grant A. R. Siegert R. K. (2018). Expert Specification of the ACMG/AMP Variant Interpretation Guidelines for Genetic Hearing Loss. Hum. Mutat. 39 (11), 1593–1613. 10.1002/humu.23630 30311386 PMC6188673

[B39] Pan Z. Xu H. Tian Y. Liu D. Liu H. Li R. (2020). Perrault Syndrome: Clinical Report and Retrospective Analysis. Mol. Genet. Genomic Med. 8 (10), e1445. 10.1002/mgg3.1445 32767731 PMC7549576

[B40] Propst E. J. Blaser S. Gordon K. A. Harrison R. V. Papsin B. C. (2005). Temporal Bone Findings on Computed Tomography Imaging in Branchio-Oto-Renal Syndrome. The Laryngoscope 115 (10), 1855–1862. 10.1097/01.mlg.0000177032.98386.20 16222209

[B41] Rickard S. Boxer M. Trompeter R. Bitner-Glindzicz M. (2000). Importance of Clinical Evaluation and Molecular Testing in the Branchio-Oto-Renal (BOR) Syndrome and Overlapping Phenotypes. J. Med. Genet. 37 (8), 623–627. 10.1136/jmg.37.8.623 10991693 PMC1734672

[B42] Ruf R. G. Xu P.-X. Silvius D. Otto E. A. Beekmann F. Muerb U. T. (2004). SIX1 Mutations Cause Branchio-Oto-Renal Syndrome by Disruption of EYA1-SIX1-DNA Complexes. Proc. Natl. Acad. Sci. 101 (21), 8090–8095. 10.1073/pnas.0308475101 15141091 PMC419562

[B43] Shah A. M. Krohn P. Baxi A. B. Tavares A. L. P. Sullivan C. H. Chillakuru Y. R. (2020). Six1 Proteins with Human Branchio-Oto-Renal Mutations Differentially Affect Cranial Gene Expression and Otic Development. Dis. Model. Mech. 13 (3), dmm043489. 10.1242/dmm.043489 31980437 PMC7063838

[B44] Shimasaki N. Watanabe K. Hara M. Kosaki K. (2004). EYA1 Mutation in a Newborn Female Presenting with Cardiofacial Syndrome. Pediatr. Cardiol. 25 (4), 411–413. 10.1007/s00246-003-0271-3 15493068

[B45] Song M. H. Kwon T.-J. Kim H. R. Jeon J. H. Baek J.-I. Lee W.-S. (2013). Mutational Analysis of EYA1, SIX1 and SIX5 Genes and Strategies for Management of Hearing Loss in Patients with BOR/BO Syndrome. PLoS One 8 (6), e67236. 10.1371/journal.pone.0067236 23840632 PMC3696009

[B46] Tarasov A. Vilella A. J. Cuppen E. Nijman I. J. Prins P. (2015). Sambamba: Fast Processing of NGS Alignment Formats. Bioinformatics 31 (12), 2032–2034. 10.1093/bioinformatics/btv098 25697820 PMC4765878

[B47] Uno T. Sawada M. Kurotaki T. Shinomiya N. (2004). EYA1 Gene Nonsense Mutation in a Japanese Family with Branchio-Oto-Renal Syndrome. Pediatr. Int. 46 (5), 615–617. 10.1111/j.1442-200x.2004.01935.x 15491396

[B48] Unzaki A. Morisada N. Nozu K. Ye M. J. Ito S. Matsunaga T. (2018). Clinically Diverse Phenotypes and Genotypes of Patients with Branchio-Oto-Renal Syndrome. J. Hum. Genet. 63 (5), 647–656. 10.1038/s10038-018-0429-8 29500469

[B49] Usami S. Abe S. Shinkawa H. Deffenbacher K. Kumar S. Kimberling W. J. (1999). EYA1 Nonsense Mutation in a Japanese Branchio-Oto-Renal Syndrome Family. J. Hum. Genet. 44 (4), 261–265. 10.1007/s100380050156 10429368

[B50] Wang S.-H. Wu C.-C. Lu Y.-C. Lin Y.-H. Su Y.-N. Hwu W.-L. (2012). Mutation Screening of the EYA1, SIX1, and SIX5 Genes in an East Asian Cohort with Branchio-Oto-Renal Syndrome. The Laryngoscope 122 (5), 1130–1136. 10.1002/lary.23217 22447252

[B51] Wang Y.-G. Sun S.-P. Qiu Y.-L. Xing Q.-H. Lu W. (2018). A Novel Mutation in EYA1 in a Chinese Family with Branchio-Oto-Renal Syndrome. BMC Med. Genet. 19 (1), 139. 10.1186/s12881-018-0653-2 30086703 PMC6081847

[B52] Xu P.-X. Adams J. Peters H. Brown M. C. Heaney S. Maas R. (1999). Eya1-deficient Mice Lack Ears and Kidneys and Show Abnormal Apoptosis of Organ Primordia. Nat. Genet. 23 (1), 113–117. 10.1038/12722 10471511

[B53] Yashima T. Noguchi Y. Ishikawa K. Mizusawa H. Kitamura K. (2003). Mutation of theEYA1Gene in Patients with Branchio-Oto Syndrome. Acta Oto-Laryngologica 123 (2), 279–282. 10.1080/0036554021000028103 12701758

[B54] Zhang Y. Knosp B. M. Maconochie M. Friedman R. A. Smith R. J. H. (2004). A Comparative Study of Eya1 and Eya4 Protein Function and its Implication in Branchio-Oto-Renal Syndrome and DFNA10. J. Assoc. Res. Otolaryngol. 5 (3), 295–304. 10.1007/s10162-004-4044-3 15492887 PMC2504552

