# Supplementary figures and images for: Genetic and Phenotypic Variability in Chinese Patients With Branchio-Oto-Renal or Branchio-Oto Syndrome
Source: Front Genet. 2021 Nov 15;12:765433. doi: 10.3389/fgene.2021.765433 (PMC8634836; doi:10.3389/fgene.2021.765433)

(A)

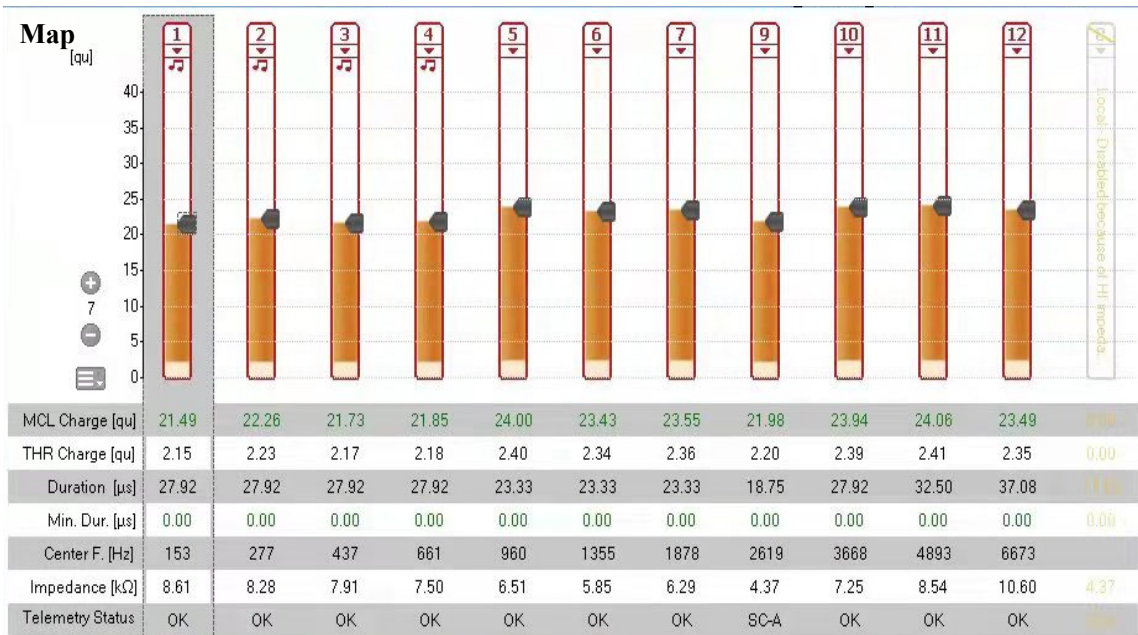

(B)

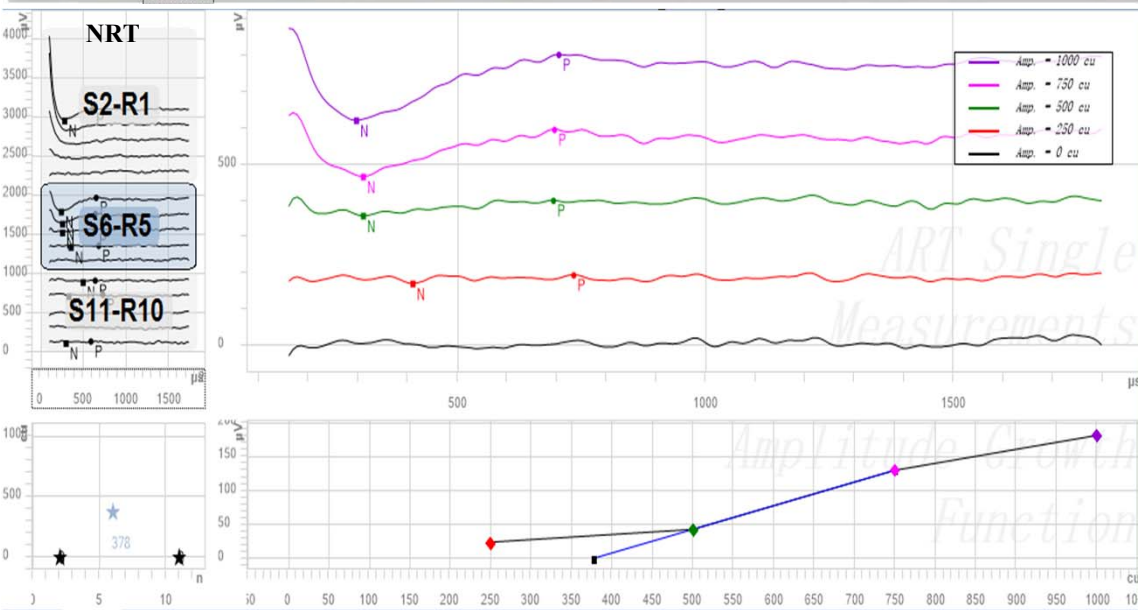

(C)

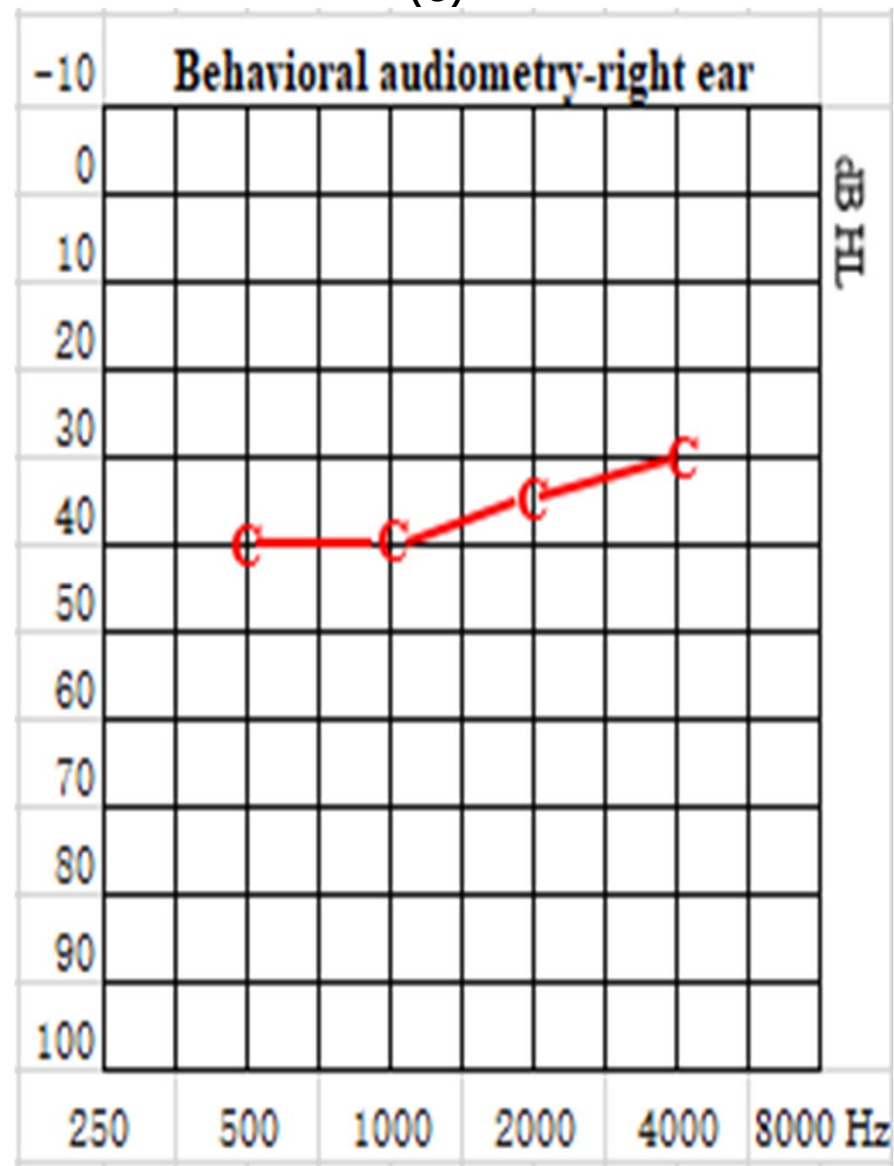

Supplement: Supplementary file 1 [file DataSheet1.PDF]
